# Exosomes in Cancer Therapy

**DOI:** 10.3390/cancers14030500

**Published:** 2022-01-20

**Authors:** Farrukh Aqil, Ramesh C. Gupta

**Affiliations:** 1 UofL Health—Brown Cancer Center, University of Louisville, Louisville, KY 40202, USA; 2 Department of Medicine, University of Louisville, Louisville, KY 40202, USA; 3 Department of Pharmacology and Toxicology, University of Louisville, Louisville, KY 40202, USA

Exosomes or small extracellular vesicles (EVs) are natural nanoparticles and known to play essential roles in intercellular communications, carrying a cargo of a broad variety of lipids, proteins, metabolites, RNAs (mRNA, miRNA, tRNA, long non-coding RNA), and DNAs (mtDNA, ssDNA, dsDNA). This is an emerging field. Recent studies have been conducted on exosomes demonstrating physical and biological stability and suitable tolerability, simplicity of preparation, possibility of commercial scale-up and functionalization for tumor-targeting. These features make exosomes ideal nanoparticles for drug delivery, with wide therapeutic applications. These findings have invigorated researchers to explore the exosomes and their roles under both physiological and pathological conditions in greater detail.

Exosomes participate in complex biological responses and have been proposed for drug-delivery purposes, as they can be loaded with both small molecules and macromolecules, which endorse their use as therapeutic tools to treat various diseases, including cancer. There have been inherent problems associated with other nanoparticle delivery systems. Thus, for exosomes to be accepted as a drug carrier in clinics, the development of biocompatible and economically viable exosomes, which are effective and well-tolerated in vivo, must be demonstrated. Exosomes have many of the desirable features, such as a long circulating half-life, the intrinsic ability to target tissues, biocompatibility, and minimal or no inherent toxicity issues, overcoming the limitations observed with the majority of other delivery systems.

In the last decade, there has been an exponential growth in the field of exosomes, with about 21,000 publications on exosomes listed in PubMed alone and over 4200 in the year 2020 (Figure 1). Due to their nano-size (30–150 nm) and biological functions, exosomes have been used as nano-carriers for small molecules and macromolecules (siRNA and pDNA) in cancer therapy in pre-clinical studies, as well as biomarkers for cancer diagnosis and prognosis. Progress in the use of exosomes in clinical studies has been slow. Kalluri’s laboratory recently reported a scalable production of exosomes from mesenchymal stem cells (MSCs) using a bio-reactor [1] and listed a clinical trial in the National Institutes of health website (NCT03608631) with the exosome-mediated delivery of si*KRAS^G12D^* against pancreatic cancer [2]. The abundance of exosomes is orders of magnitude higher in bovine milk [3] and colostrum powder [4,5] compared to cell culture media. In this regard, the research article by Kandimalla and colleagues [5] presented in this Special Issue showed the utility of exosomes isolated from bovine colostrum powder for delivery of the therapeutic drug paclitaxel, which is of high clinical relevance. This article highlights the tumor targetability of exosomes and successfully showed that an oral functionalized exosomal formulation of paclitaxel significantly improved the efficacy and mitigated immunotoxicity, while providing a user-friendly, cost-effective alternative to an intravenous bolus dose standard-of-care paclitaxel and abraxane. Reviewing the progress from the discovery to the therapeutic development of exosomes, Jan et al. [6] summarized the valuable information on exosome donor cell types, exosome cargoes, cargo loading, routes of exosome administration, and the engineering of exosomal surfaces for specific peptides that increase target specificity and, as such, therapeutic efficacy.

Exosomes derived from cancer cells carry the cargo reflective of genetic alterations in cancer cells and could likely serve as a biomarker in the early detection of cancer. To further highlight this area of exosome research, Guerrini and colleagues [7] discuss the role of exosomes as one of the most intriguing cancer biomarkers in modern oncology for early cancer diagnosis, prognosis and treatment monitoring. They discussed the application of plasmonic devices exploiting surface-enhanced Raman spectroscopy (SERS) as the optosensing technique for the structural interrogation and characterization of the heterogeneous nature of exosomes. Using a similar concept, Bondhopadhyay and colleagues [8] reviewed the role of exosomes in communication between tumor cells in the breast cancer tumor microenvironment. They highlighted the role of exosomes in breast carcinogenesis and how exosomes could be used or targeted by recent immunotherapeutics to achieve promising intervention strategies.

The role of Rab proteins and endocytosis process was discussed by Sinha et al. [9]. In this article, authors reviewed the potential of exosomes in many aspects of cancer biology including exosome biogenesis, cargo, Rab-dependent and Rab-independent secretion of endosomes and exosomal internalization. They further show that exosomes could migrate to distal parts and propagate oncogenic signaling and epigenetic regulation, modulate the tumor micro-environment and facilitate the immune escape, tumor progression and drug resistance responsible for cancer progression. The exosomes have been detected in all the bodily fluids, and play different roles based on their origin. In the research manuscript, Cacic and colleagues [10] showed that platelet-derived microparticles were internalized by THP-1 cells, and resulted in increased levels of different miRNAs such as miR-125a, miR-125b, and miR-199.

Recent advances have confirmed exosomes immunotherapy as a feasible, safe option leading to both innate and adaptive immune responses. Exosomes possess different properties according to their source and the cargo they carry. The review by Giacobino et al. [11], provides an up-to-date summary of exosome use in cancer immunotherapy involving the use of exosomes to transport molecules that are able to trigger an immune response and damage cancer cells. Furthermore, besides basic notions regarding cancer immunotherapy, this article focuses on the potential of exosome-based therapeutic vaccines in the treatment of cancer patients, overviewing the clinically relevant trials. This approach may represent a potential target for future anti-cancer therapy. On a similar line, Yao et al. [12] reviewed dendritic-cell-derived exosome vaccines exhibiting better antitumor efficacy in pre-clinical animal models. This review further highlights recent clinical trials with DC exosomes as cancer vaccines and discuss why they only showed limited clinical efficacy in advanced cancer patients. Since clinical studies failed to induce tumor-specific T-cell responses, these observations could be helpful in future clinical studies on the fight against cancer.

Tumor cell exosomes have also been shown to contain danger-associated molecular patterns (DAMPs), which are released in response to cellular stress to alert the immune system to the dangerous cell. Linder and Strandmann [13] shed the light on this aspect in their review. They discuss the role of exosomes in the defense mechanism of heat shock protein 70 (HSP70), as HSP70-positive T-EVs are known to trigger anti-tumor immune responses. The release of DAMPs, including HSP70, may also induce chronic inflammation or suppress immune cell activity, promoting tumor growth. They summarize the current knowledge on soluble, membrane-bound, and EV-associated HSP70 regarding their functions in regulating tumor-associated immune cells in the tumor microenvironment. Additionally, a valuable discussion on the immunotherapies that aimed to target HSP70 and its receptors for cancer treatment is presented.

As discussed above, while exosomes have shown utility in the diagnosis, and treatment of various cancers, recent evidence reveals that cancer-cell-derived exosomes can change the behavior of target cells. The review represented by Burgos-Ravanal [14] discussed that exosomes isolated from aggressive cancer cells can transfer their “traits” to less aggressive cancer cells and convert them into more malignant tumor cells. This review further highlights the role of exosomes in drug resistance, and provides a valuable discussion on why pharmacological therapies are often ineffective. Besides highlighting how inhibiting exosome production could interfere in reduced metastasis and drug resistance, this review highlights exosomes that can be used for therapeutic and prognostic purposes in cancer.

In summary, this Special Issue comprises informative research and authoritative review articles written by an international group of expert scientists and comprehensively discusses exosome biogenesis and protein-sorting, the isolation of exosomes, better loading efficiency, and targeted delivery of drugs and their roles in cancer diagnosis, progression, metastasis and treatment. The articles represent preclinical in vitro and in vivo data to demonstrate exosomes as an oral delivery vehicle for cancer therapeutics. Various reviews debate/discuss strategies where the use of exosomes led to ‘cheerful’ results when exploring diagnosis and treatment options for different human cancers.

## Figures and Tables

**Figure 1:**
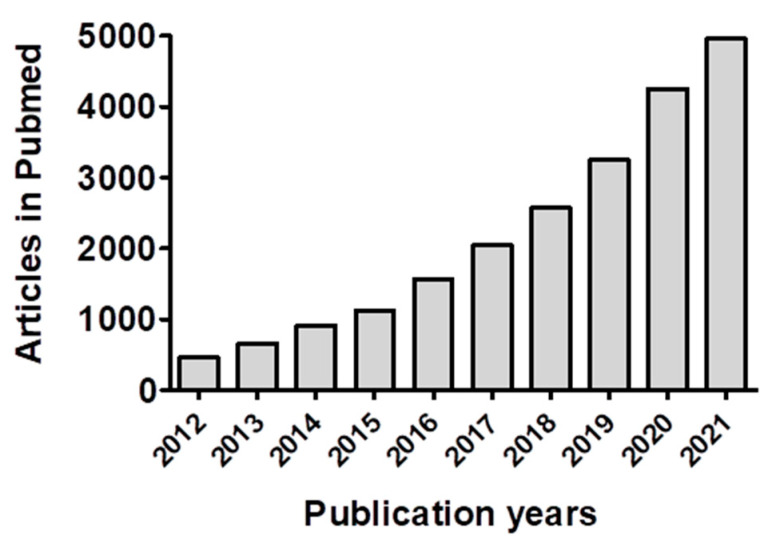
List of publications on exosomes in PubMed in last 10 years.
